# Expression of concern: Glucose sensing by a glassy carbon electrode modified with glucose oxidase and a magnetic polymeric nanocomposite

**DOI:** 10.1039/d5ra90053k

**Published:** 2025-05-02

**Authors:** Mehdi Baghayeri

**Affiliations:** a Department of Chemistry, Faculty of Science, Hakim Sabzevari University PO Box 397 Sabzevar Iran m.baghayeri@hsu.ac.ir +98 5144013170 +98 5144013325

## Abstract

Expression of concern for ‘Glucose sensing by a glassy carbon electrode modified with glucose oxidase and a magnetic polymeric nanocomposite’ by Mehdi Baghayeri, *RSC Adv.*, 2015, **5**, 18267–18274, https://doi.org/10.1039/C4RA15888A.

*RSC Advances* is publishing this expression of concern in order to alert readers that concerns have been raised over the integrity of the data published in Fig. 2 in this article. The author has been contacted but has not responded to requests to provide raw data. An expression of concern will continue to be associated with the article until a conclusive outcome is reached.

Laura Fisher

25^th^ April 2025

Executive Editor, *RSC Advances*

